# Sarcoidosis: a general overview

**DOI:** 10.1186/s42358-024-00381-z

**Published:** 2024-08-12

**Authors:** Fabricio Souza Neves, Ivanio Alves Pereira, Flavio Sztajnbok, Nilton Salles Rosa Neto

**Affiliations:** 1 Internal Medicine Department, Rheumatology Division, Health Sciences Center, Universidade Federal de Santa Catarina (UFSC), Florianópolis, Brazil; 2 Medicine School, Rheumatology Division, Universidade do Sul de Santa Catarina (UNISUL), Palhoça, Brazil; 3 Department of Pediatrics, Universidade Federal do Rio de Janeiro, Rio de Janeiro, Brazil; 4 Centro de Doenças Raras e da Imunidade, Hospital Nove de Julho, São Paulo, Brazil; 5 Universidade Santo Amaro, São Paulo, Brazil

**Keywords:** Sarcoidosis, Rare diseases, Review, Noncaseating granuloma

## Abstract

Sarcoidosis is a systemic inflammatory disease of unknown origin, which consists of the formation of multiple sterile noncaseating granulomas. Inhaled antigens are believed to initiate disease in prone individuals, considering that almost all patients present pulmonary or mediastinal lymph node disease. Extrapulmonary manifestations are common and diverse: practically any organ system can be affected, and treatment can range from simple watchful waiting to intense immunosuppression. In this article, we review current concepts about sarcoidosis in an overview, focusing on recognition and treatment of its major clinical phenotypes.

## Background

Sarcoidosis is a systemic inflammatory disease of unknown origin. It is more common in Western countries and typically occurs in adults. It affects the lungs and mediastinal lymph nodes in almost all cases, but it can also involve the heart, skin, kidneys, eyes, peripheral and central nervous system, and joints. Biopsy of affected sites remains the cornerstone of the diagnosis, given that its main characteristic is the pathologic pattern based on noncaseating granulomas [1]. Although many cases may be self-limiting, some patients require immunosuppressive therapy to prevent end-organ damage or even death due to failure of vital organs, particularly heart and lungs. The incidence and severity of the disease appears to be higher in people of African descent living in the United States [1, 2].

## Pathogenesis and pathology

Like many other idiopathic inflammatory diseases, sarcoidosis is believed to occur in individuals with certain genetic predispositions after exposure to environmental triggers. However, both factors are essentially unknown [1]. Sarcoidosis is associated with some human leukocyte antigens (HLA) class II alleles, but not with a specific genetic variant [2, 3]. In granulomatous lesions of sarcoidosis, different microbial DNA was found, but viable microorganisms were never observed [4]. Also exposure to inorganic dusts [5] and immunoreactivity against these compounds [6] are associated with sarcoidosis. Therefore, sarcoidosis is neither a genetic nor an infectious disease; it is probably a disease that arises in individuals who develop an inappropriate immune response when challenged by certain infectious or inorganic antigens. This inappropriate immune response is based on the Th1 and Th17 pathways and induces the granulomatous inflammatory process typical of sarcoidosis, in which tumour necrosis fator alpha (TNFa) produced by activated macrophages has a fundamental role (Table 1) [7].

**Table 1:** HLA alleles linked to sarcoidosis risk, sources of antigens found in sarcoid lesions and characteristics of immune response of sarcoidosis (adapted from Greaves et al, 2020) [7]

HLA alleles	DRB1*12:01, *11:01, *03:01 DQA1*06:02 DQB1*05:03, *06:04
Source of antigens	*Mycobacterium tuberculosis; Cutibacterium acne ; Borrelia burgdorferi; Aspergillus fumigatus* Silica; berilium
Immune response	Th1: IFNg and IL-2 Th17: IL-17 and IL-23 Macrophages and epithelioid cells: TNFa

An interesting hypothesis is that the granulomatous response persists as long as there is insufficient clearance of the causative antigen, and throughout this period it causes different symptoms to the patient (it could explain the habitual self-limited course of the disease). In this hypothesis, the entry of the causative antigens is possibly via inhalation, an idea that can be deduced from the fact that almost all cases of sarcoidosis begin with pulmonary involvement and mediastinal lymphadenopathy. Biopsies of organs affected by sarcoidosis reveal multiple granulomas, which consist of clusters of multinucleated giant cells and epithelioid cells, organized in a round shape, surrounded by lymphocytes. Staining for infectious agents (at least mycobacteria and fungi) must be negative. In the center of these granulomas there is generally no necrosis [8]. For this reason, they are usually described as noncaseating granulomas, as opposed to the granuloma typical of infectious diseases, which often reveals extensive necrotic areas in its center, presenting a macroscopic “cheese-like” (caseating) appearance. However, mature granulomas in sarcoidosis also may present central areas of fibrinoid necrosis, and there are reports of extensive necrosis in sarcoid granulomas described as “caseating” in some areas. Thus the finding of necrosis or even some “caseum” at biopsy is not sufficient to rule out the diagnosis of sarcoidosis [9].

## Epidemiology

Sarcoidosis is a worldwide disease, but the frequency of diagnosis is unevenly distributed. The annual incidence is highest in Northern Europe (15 cases / 100,000 inhabitants) and North America (10/100,000), while it is rare in Eastern Asia and in the Southern Hemisphere (less than 1/100,000). The reasons for this distribution are unknown. In the United States, the incidence in the African American population (18/100,000) is approximately twice that in the white American population (8/100,000), and this is approximately twice that in the American Latino population (4/100,000) and Asian-American (3/100,000). Again, the reasons for these differences are unknown [1, 9]. Sarcoidosis incidence is approximately the same in men and women and the average age at the diagnosis is around 50 years [1].

## General clinical features and approach to diagnosis

Almost any organ can be affected by sarcoidosis. Skin (14–16%), eyes (8–20%), liver (11–18%), spleen (7–20%), heart (2–11%), joints (8–9%), nervous system (2–7%), parotid glands (4%) and kidneys (2%) are common sites of sarcoid disease. However, lungs and mediastinal lymph nodes are by far most affected (> 90% of patients) [10, 11]. Thus, respiratory symptoms and abnormalities on chest radiography (CXR) or computed tomography (CT) are the most frequent initial clinical picture. If sarcoidosis is suspected in any organ system, respiratory symptoms must be inquired, and CXR and CT obtained. Chest radiography in sarcoidosis is still classified according to the staging system proposed by Scadding in 1961, with prognostic implications (Table 2) [12]. Thoracic CT has greater sensitivity and interobserver reliability than CXR and increases the accuracy of diagnosing sarcoidosis [13]. Typical findings in pulmonary parenchyma are micronodules with perilymphatic distribution in the upper and middle zones (active sites of granulomatous inflammation), ground-glass opacities, interlobular septal thickening and finally fibrotic alterations with septal bands, bullae, honeycombing, traction bronchiectasis, and loss of lung volume. Respiratory symptoms can occur in any stage of the disease but are not universal. Cough (productive or non-productive) is the most common symptom (up to 50% of patients). Dyspnea may occur in later stages of disease and wheezing may appear due to airway reactivity to inflammation. Chest pain and hemoptysis are less common. Hence, almost half of patients can be asymptomatic, reinforcing the role of chest imaging.

**Table 2:** Chest radiography staging for pulmonary sarcoidosis (adapted from Scadding, 1961) [12]

Stage	Radiographic findings
0	Normal
I	Only hilar lymphadenopathy
II	Hilar lymphadenopathy and parenchymal abnormality
III	Only parenchymal abnormality
IV	Pulmonary fibrosis with volume loss

Regardless of the presence or absence of respiratory symptoms, almost half of patients with sarcoidosis have extrapulmonary findings. Two clinical syndromes have high specificity for sarcoidosis and its recognition is useful for diagnosis. Löfgren syndrome, which is defined by acute onset of erythema nodosum and ankle arthritis with hilar adenopathy at CXR, occurs in up to 10% of patients [14]. Heerfordt syndrome is a rare presentation consisting of acute bilateral parotid gland enlargement, anterior uveitis, and facial nerve palsy [15]. In these two clinical scenarios, the diagnosis of sarcoidosis can be defined without assessing mediastinal lymph nodes for biopsy. Caution must be taken, however, in locations with high prevalence of tuberculosis, which also can cause mediastinal lymph node enlargement and erythema nodosum mimicking the Löfgren syndrome (tuberculin skin test and interferon gamma release assay are useful to assess the possibility of tuberculosis in these cases). For all other clinical presentations, a biopsy of an affected tissue revealing typical sarcoid granuloma should be done.

A particular situation occurs in cases where sarcoidosis is suspected in a clinically asymptomatic patient, where a CXR was ordered for another reason (trauma, periodic examination, etc.) and enlarged mediastinal lymph nodes were found. In these cases, expectant observation may be an appropriate approach, considering that a significant amount of sarcoidosis cases progress to spontaneous remission. However, if this is the case, monitoring the patient with imaging tests must be rigorous due to the possibility of lymphoma as a differential diagnosis. The appearance of symptoms or further increase in lymph node size would indicate the inevitability of lymph node biopsy.

Unless there is an easily accessible affected organ (e.g., skin, parotid gland), mediastinal lymph nodes or lung parenchyma are usually chosen for biopsy, which can be performed through endobronchial ultrasound-guided transbronchial needle aspiration [16]. During this procedure, a bronchoalveolar lavage is usually performed for the differential diagnosis of infectious granulomatous disease (the exclusion of these diseases is an essential part of the diagnostic workup for sarcoidosis). The typical finding of sarcoidosis is a sterile lavage revealing lymphocytosis with elevated CD4/CD8 ratio > 3.5 and T cells expressing the Vα2.3 receptor > 10.5% [17, 18]. Other approaches to the lymph nodes or pulmonary parenchyma (mediastinoscopy or thoracoscopic biopsy) may be the choice according to the clinical presentation of the disease. If an affected site is not easily identified, 18-fluorodeoxyglucose-positron emission tomography (PET-CT) may be helpful in detecting lesions that are likely to be biopsied [19].

Serum biomarkers (serum angiotensin converting enzyme, soluble interleukin-2 receptor (sIL-2R)) have limited diagnostic accuracy and they are not substitutes for biopsy [20]; Erythrocyte sedimentation rate and C-reactive protein, as markers of systemic inflammation, are usually elevated in active sarcoidosis. Patients may complain of nonspecific symptoms of inflammation, such as fever and fatigue.

## Clinical phenotypes of sarcoidosis and treatment

### Pulmonary and mediastinal lymph node sarcoidosis

Pulmonary and lymph node sarcoidosis is the most common sarcoidosis phenotype and serves as a basic reference for the treatment of sarcoidosis in general. Asymptomatic patients with stage I, or even II or III CXR, can be initially monitored without treatment, because most asymptomatic patients with sarcoidosis falling in these categories will progress to spontaneous remission. A reasonable approach is to assess symptoms, radiographic progression and pulmonary function tests results (ideally including carbon monoxide diffusion) every 3 to 6 months.

For symptomatic patients, for those with severe disease (CXR stage IV) and for those with progressive disease under follow-up, treatment should be offered.

First-line treatment is based on oral glucocorticoids (prednisone 20–40 mg/day). In a periodic follow-up of 3 to 6 months, improvement in the disease would allow the clinician to taper the dose until withdrawal or reaching the lowest dose that provides satisfactory relief of symptoms and control of the disease. A second line (usually methotrexate) and a third line (usually mycophenolate) non-biologic agents can be used if glucocorticoids monotherapy was not effective. Hydroxychloroquine can be added in combination with other non-biologic agents. Failure of non-biologic treatment should lead to the prescription of anti-TNF treatment (there is greater evidence on infliximab at a dosage of 5 mg/kg, with the usual loading dose at 0, 2 and 6 weeks) [21]. Efzotimimod, a Fc fusion protein that binds to neuropilin-2 (NRP-2) highly expressed in sarcoid granulomas, has been evaluated in refractory pulmonary sarcoidosis with promising results [22].

### Sarcoid arthropathy

Löfgren syndrome is the most common presentation of acute sarcoid arthropathy. More than half of patients will progress to spontaneous remission (erythema nodosum for up to six months, but joint pain may persist up to 2 years) especially those who are positive for HLA-DRB1*03 [23, 24]. Arthritis is usually oligoarticular and commonly affects the knees, ankles, elbows, wrists, and small joints of the hands and feet [25]. Due to the self-limiting nature of this condition, treatment is symptomatic and can be based in the use of nonsteroidal antiinflammatory agents with or without low-dose prednisone (≤ 10 mg/day). Less than a third of patients will evolve to persistent disease and require treatment similar to that of chronic sarcoid arthropathy [14].

Chronic sarcoid arthropathy is uncommon and can present as a polyarthritis in a rheumatoid-like pattern (proximal interphalangeal, metacarpophalangeal, hands, wrists, knees, and ankles). Jaccoud deformities and bone erosion may occur in the course of disease. In rare cases, Synovial biopsies reveal typical noncaseating granulomas, but they are rarely necessary for the diagnosis, which is generally made by combining the recognition of typical chronic polyarthritis in a patient with an already confirmed diagnosis of pulmonary and lymph node sarcoidosis, and the exclusion of the possibility of rheumatoid arthritis. In situations of diagnostic doubt that require synovial biopsy, arthroscopy-guided biopsies should be preferred, as blind punctures have lower diagnostic yield.

Patients with chronic sarcoid arthropathy should receive hydroxichloroquine in combination with low-dose glucocortidoids. Failure of this treatment should lead to the association of methotrexate as a second-line agent and leflunomide as a third-line agent. Failure to non-biologic treatment should prompt the prescription of anti-TNF agents. There is evidence on the use of infliximab, at a dosage of 5 mg/kg every four to eight weeks [26].

### Ocular sarcoidosis

The eyes and surrounding structures may be affected in about a quarter of patients with sarcoidosis. Uveitis, keratoconjunctivitis sicca and ocular adnexal granulomas are the most common presentations [27]. Screening for ocular sarcoidosis by an ophthalmologist is recommended at the time of the diagnosis (baseline), and ophthalmologic examination should be performed whenever ocular symptoms appear during follow-up [28].

Sarcoid uveitis can be anterior, intermediate, or posterior. It is usually bilateral and has a good prognosis [29]. Anterior uveitis typically causes pain and redness in the limbus (boundary between the cornea and sclera); posterior or intermediate uveitis is usually painless, more often associated only with the patient perception of visual floaters. Slit-lamp examination can reveal intraocular signs suggestive of sarcoid uveitis, which can define the diagnosis in a patient with previously established diagnosis of pulmonary or lymph node sarcoidosis [30]. Optical coherence tomography of the macular region to exclude macular edema, or fluorescein angiography to rule out papillitis and/or vasculitis or ischemia of the retina can be done. In rare cases, anterior chamber biopsy or conjunctival biopsy may be needed to investigate a doubtful case. Anterior uveitis can be treated with topical glucocorticoids. However, refractory cases, intermediate and posterior uveitis need first-line treatment with high-dose systemic glucocorticoids (prednisone 40–60 mg/day). Sarcoidosis also may affect extraocular orbital tissues, including the lacrimal glands (leading to keratoconjunctivitis sicca), the conjunctiva and extraocular muscles, where it can present as an orbital soft tissue mass. These lesions are also usually treated with systemic glucocorticoids.

Failure to this treatment should lead to the prescription of second-line therapy with methotrexate or azathioprine. A second failure should lead to the use of a calcineurin inhibitor (cyclosporine or tacrolimus) or mycophenolate. After that, anti-TNF agents should be used. Periocular and intravitreal injections of glucocorticoids or long-acting glucocorticoids implants can be added to the therapy before progression of systemic immunosuppression. Symptomatic therapy to minimize ocular dryness in keratoconjunctivitis sicca should be used whenever necessary [31].

### Cutaneous sarcoidosis

Skin lesions occur in approximately one-quarter of patients with sarcoidosis. The skin lesions of sarcoidosis are easily accessible for biopsy, facilitating the diagnostic work in a suspected case, eventually eliminating the need for a mediastinal lymph node biopsy. However, the most common skin lesion appearing in sarcoidosis, erythema nodosum, is nonspecific for the diagnosis of sarcoidosis. Its pathology reveals only septal paniculitis (without granulomas), as occurs in many other diseases that manifest erythema nodosum. The typical noncaseating granulomas are only seen in the so-called specific sarcoid cutaneous lesions: papular sarcoidosis (non-scaly papules occuring on the face, mainly on the eyelids and nasolabial folds); plaque sarcoidosis (more common on the trunk, especially the shoulders and back); nodular sarcoidosis (a collection of granulomas grouped in dermis or subcutaneous fat tissue) and the lupus pernio (infiltrative erythematous plaques distributed on the central face and surroundings) [32]. Many variations, subtypes and combinations of these major cutaneous specific lesions may occur in sarcoidosis, and treatment may vary according to the predominant skin feature and its severity. Lupus pernio is usually resistant to traditional sarcoidosis treatment and may require anti-TNF therapy more frequently than other lesions [25, 33].

### Cardiac sarcoidosis

Sarcoidosis affects the heart in up to 30% of patients, but the prevalence may be higher, such as 50%, as shown in Japanese post-mortem studies [34]. Considering the risk of death, it is important to screen for cardiac involvement in all patients with sarcoidosis. Patients may experience palpitations, syncope, chest pain or even sudden death. Atrioventricular blocks and ventricular arrhythmias are more frequent, but ventricular tachycardia and supraventricular arrhythmia also occur. Heart failure with reduced ejection fraction due to dilated cardiomyopathy and heart failure with preserved ejection fraction due to restrictive cardiomyopathy are observed. Echocardiography can show abnormalities in longitudinal strain patterns detecting subclinical myocardial dysfunction, or regional wall movements that do not correspond to the territory of coronary arteries [35, 36].

Endomyocardial biopsy shows granulomatous inflammation of the myocardium on histopathology, but the procedure is associated with considerable risk of complications and has low sensitivity due to the irregular distribution of granulomas in cardiac tissues. Therefore, the diagnosis of cardiac involvement relies heavily on cardiac imaging studies. Both gadolinium-enhanced cardiovascular magnetic resonance (MRI) and 18 F-fluorodeoxyglucose PET-CT are useful for diagnosing cardiac sarcoidosis [34, 37]. The characteristic MRI pattern is multifocal areas of subepicardial and mid-myocardial late gadolinium enhancement, which is an indicator of fibrosis. Late gadolinium enhancement is typically seen in the basal segments of the septum and lateral wall, although more extensive involvement can be seen, including the right ventricle [25].

Treatment of cardiac sarcoidosis begins with prednisone 40–60 mg/day, but methotrexate can be added. The use of anti-TNF drugs such as infliximab and adalimumab is a good option in severe cases or patients who have failed treatment. For patients with severe atrioventricular blocks, pacemakers are used along with implantable cardioverter defibrillators for patients with low ejection fraction.

### Neurological sarcoidosis

The nervous system is affected in 5–10% of sarcoidosis patients. Any part of the central or peripheral nervous system can be involved, but cranial peripheral neuropathy is particularly common (almost half of neurosarcoidosis patients have some form of cranial peripheral neuropathy, mostly facial nerve palsy). Meningeal, cerebral, hypothalamic/hypophyseal, spinal cord and radicular dysfunctions may occur due to perivascular formation of sarcoid granulomas. Peripheral axonal, demyelinating, and small fiber neuropathies have also been described. Diagnosis is based on clinical, imaging, neurophysiological examinations and analysis of cerebrospinal fluid, according to each case. Neurological biopsy is generally unnecessary when sarcoidosis occurs in another, more easily accessible organ system. When involvement of other organic systems is not clinically evident, 18 F-fluorodeoxyglucose PET-CT may be useful in searching for potential biopsy sites.

Treatment of neurological sarcoidosis is also based, primarily, on glucocorticoids. Very high dosages (intravenous methylprednisolone 20 mg/kg/day for 3 days) can be used for life-threatening or rapidly progressive neurological disease. Second-line therapy also consists of non-biological immunosuppressive therapy (mycophenolate, azathioprine, methotrexate, cyclophosphamide, and leflunomide have been used). Third-line therapy comprises biologic therapy. Again, there is more evidence with anti-TNF agents (mostly infliximab). Radiation therapy may also be considered for refractory lesions [38, 39].

### Renal sarcoidosis

The kidneys may be indirectly affected in sarcoidosis due to hypercalcemia. Almost 10% of sarcoidosis patients present with high serum calcium concentration due to extrarenal production of calcitriol by activated macrophages, leading to hypercalciuria that can induce nephrocalcinosis and urolithiasis. Chronic renal failure may develop in untreated, complicated cases [40]. In addition to controlling sarcoidosis, treatment includes increasing the solubility of urinary calcium phosphate or calcium oxalate. Kidneys can also be directly damaged by sarcoidosis from tubulointerstitial sarcoid chronic nephritis. Biopsies of this condition reveal the typical noncaseating granulomas between normal renal glomeruli, but it rarely leads to renal failure, thus aggressive immunosuppressant treatment usually is not required [41].

### Gastrointestinal and abdominal sarcoidosis

Sarcoidosis can affect any part of the gastrointestinal tube, liver, pancreas, spleen, and peritoneum, although it is considered as a rare phenotype of the disease. Definitive diagnosis of sarcoidosis in this context can be difficult, because intestinal non-caseating granulomas can have more frequent etiologies, as Crohn’s disease. In addition to gastrointestinal biopsy, it is recommended to check the occurrence of sarcoidosis in another organic system, increasing the probability of diagnosis [42].

Symptoms associated with intra-abdominal sarcoidosis depend on the affected site and may range from visible lesions (oral cavity), dysphagia (esophagus), occult bleeding and diarrhea (stomach and intestine), abdominal pain, among others. All intra-abdominal forms of sarcoidosis are considered rare, except for the occurrence of intrahepatic granulomas, which is present in almost half of patients with sarcoidosis undergoing liver biopsy and is generally asymptomatic. There is no consensus regarding the indication for treatment. Treatment (glucocorticoids as first line therapy) is normally done in symptomatic cases or when there is a significant increase in the serum concentration of hepatic or canalicular enzymes, particularly to those cases with intrahepatic biliary strictures due to sarcoid granulomas [43].

### Pediatric sarcoidosis

The sarcoidosis described in this article (a disease of multifactorial etiology) may affect the pediatric population, although rarely. When this happens, the disease usually occurs in adolescents and older children similarly to adult cases (frequent involvement of the lungs and mediastinal lymph nodes associated to several other systemic manifestations) and is called pediatric-onset adult-type sarcoidosis [44]. In younger children, systemic granulomatous inflammatory disease is most frequently caused by one of numerous possible gain-of-function variants of NOD2 (nucleotide-binding oligomerization domain-containing protein 2) and generally presents without lung or lymph node involvement. Arthritis is the most common manifestation, sometimes with the triad of polyarthritis, erythematous rash and uveitis, mimicking systemic juvenile arthritis. This condition is called Blau syndrome/Early Onset Sarcoidosis and can be recognized by typical family history (autosomal dominant inheritance) or appear sporadically with a de novo variant. Despite the name and the typical non-caseating granulomas on biopsy, it should be considered an entity distinct from adult-type sarcoidosis. Pathogenic NOD2 variants have not been observed in adult-type sarcoidosis and clinical presentation of both diseases are quite different. There is similarity in treatment (mainly based on glucocorticoids, non-biologic immunosuppressant agents and anti-TNF biologic therapy), but evolution in adult-type sarcoidosis is for remission in nearly half of cases, while Blau syndrome/early onset sarcoidosis is, with currently available treatments, an inevitably permanent disease that usually requires lifelong immunosuppressive therapy [45].

## Conclusion

A rheumatologist can obviously treat and help people with sarcoid arthropathy, but not only this. It is even more common for a rheumatologist to be asked in a suspected case of sarcoidosis to act as a clinician experienced in the investigation and treatment of rare systemic inflammatory disorders in general, not focusing on a single organ system. For this reason, rheumatologists must be aware of the several phenotypes of sarcoidosis, including the distinct Blau syndrome/early onset sarcoidosis of young children, collaborating with the focal specialists appropriate to each case.

## Data Availability

Not applicable.

## References

[1] Rossides M, Darlington P, Kullberg S, Arkema EV. Sarcoidosis: Epidemiology and clinical insights. J Intern Med. 2023;293:668–80. 10.1111/joim.13629. 36872840 10.1111/joim.13629

[2] Levin MA, Adrianto I, Datta I, et al. Association of HLA-DRB1 with Sarcoidosis susceptibility and progression in African americans. Am J Respir Cell Mol Biol. 2015;53:206–16. 10.1165/rcmb.2014-0227OC. 25506722 10.1165/rcmb.2014-0227OC PMC4566045

[3] Sikorova K, Osoegawa K, Kocourkova L, et al. Association between sarcoidosis and HLA polymorphisms in a Czech population from Central Europe: focus on a relationship with clinical outcome and treatment. Front Med. 2023. 10.3389/fmed.2023.1094843. 10.3389/fmed.2023.1094843 PMC10160604 37153085

[4] Inaoka PT, Shono N, Kamada M, Espinoza JL. Host-microbe interactions in the pathogenesis and clinical course of sarcoidosis. J Biomed Sci. 2019;26:45. 10.1186/s12929-019-0537-6. 31182092 10.1186/s12929-019-0537-6 PMC6558716

[5] Graff P, Larsson J, Bryngelsson IL, Wiebert P, Wihlborg P. Sarcoidosis and silica dust exposure among men in Sweden: a case-control study. BMJ Open. 2020;10(9):e038926. 10.1136/bmjopen-2020-038926. 32883739 10.1136/bmjopen-2020-038926 PMC7473614

[6] Beijer E, Meek B, Bossuyt X, et al. Immunoreactivity to metal and silica associates with sarcoidosis in Dutch patients. Respir Res. 2020;21:141. 10.1186/s12931-020-01409-w. 32513159 10.1186/s12931-020-01409-w PMC7282065

[7] Greaves AS, Atif SM, Fontenot AP. eCollection. Adaptive Immunity in Pulmonary Sarcoidosis and Chronic Beryllium Disease. Front Immunol 2020:11:474. 10.3389/fimmu.2020.00474. 2020. 10.3389/fimmu.2020.00474 PMC7093490 32256501

[8] Cooper D, Suau S, Sarcoidosis. Immunol Allergy Clin N Am. 2023;583–91. 10.1016/j.iac.2022.10.011. 10.1016/j.iac.2022.10.011 37394261

[9] Binesh F, Halvani H, Navabii H. BMJ Case Rep. 2012. 10.1136/bcr.05.2011.4278. bcr0520114278. 22665906 10.1136/bcr.05.2011.4278 PMC3279652

[10] Te HS, Perlman DM, Shenoy C, et al. Clinical characteristics and organ system involvement in sarcoidosis: comparison of the University of Minnesota Cohort with other cohorts. BMC Pulm Med. 2020;20:155. 10.1186/s12890-020-01191-x. 32487134 10.1186/s12890-020-01191-x PMC7268634

[11] Shupp JC, Freitag-Wolf S, Bargagli E et al. Phenotypes of organ involvement in sarcoidosis. Eur Resp J 2018 51: 1700991. 10.1183/13993003.00991-2017. 10.1183/13993003.00991-2017 29371378

[12] Scadding JG. Prognosis of intrathoracic sarcoidosis in England. A review of 136 cases after five years’ observation. Br Med J. 1961;2:1165–72. 10.1136/bmj.2.5261.1165. 14497750 10.1136/bmj.2.5261.1165 PMC1970202

[13] Levy A, Hamzeh N, Maier LA, F1000Res. Is it time to scrap Scadding and adopt computed tomography for initial evaluation of sarcoidosis?. 2018; 7: F1000 Faculty Rev-600. 10.12688/f1000research.11068.1. 10.12688/f1000research.11068.1 PMC5958314 29946423

[14] Maña J, Gómez-Vaquero C, Montero A, et al. Löfgren’s syndrome revisited: a study of 186 patients. Am J Med. 1999;107:240–2455. 10.1016/s0002-9343(99)00223-5. 10492317 10.1016/s0002-9343(99)00223-5

[15] Denny MC, Fotino AD. The Heerfordt-Waldenström syndrome as an initial presentation of sarcoidosis. Proc (Bayl Univ Med Cent). 2013;26:390. 10.1080/08998280.2013.11929014. 24082416 10.1080/08998280.2013.11929014 PMC3777100

[16] Crombag LMM, Moij-Kalverda K, Szlubowski A, et al. EBUS versus EUS-B for diagnosing sarcoidosis: the International Sarcoidosis Assessment (ISA) randomized clinical trial. Respirology. 2022;27:152–60. 10.1111/resp.14182. 34792268 10.1111/resp.14182 PMC9299594

[17] Darlinton P, Kullberg S, Eklund A, et al. Lung CD4 + Vα2.3 + T-cells in sarcoidosis cohorts with Löfgren’s syndrome. Respir Res. 2020;21:61. 10.1186/s12931-020-1327-0. 32111204 10.1186/s12931-020-1327-0 PMC7048083

[18] Shen Y, Pang C, Wu Y et al. Diagnostic performance of Bronchoalveolar Lavage Fluid CD4/CD8 ratio for sarcoidosis: a Meta-analysis. EBioMedicine 2016:8:302–8. 10.1016/j.ebiom.2016.04.024. 10.1016/j.ebiom.2016.04.024 PMC4919536 27428439

[19] Costabel U, Bonella F, Ohshimo S, Guzman J. Diagnostic modalities in sarcoidosis: BAL, EBUS, and PET. Semin Respir Crit Care Med. 2010;31:404. 10.1055/s-0030-1262207. 20665390 10.1055/s-0030-1262207

[20] Ramos-Casals M, Retamozo S, Sisó-Almiral A, et al. Clinically-useful serum biomarkers for diagnosis and prognosis of sarcoidosis. Expert Rev Clin Immunol. 2019;15:391–405. 10.1080/1744666X.2019.1568240. 30632406 10.1080/1744666X.2019.1568240

[21] Rahaghi FF, Baughman RP, Saketkoo LA, et al. Delphi consensus recommendations for a treatment algorithm in pulmonary sarcoidosis. Eur Respir Rev. 2020;29:190146. 10.1183/16000617.0146-2019. 32198218 10.1183/16000617.0146-2019 PMC9488897

[22] Baughman RP, Niranjan V, Walker G, et al. Efzofitimod: a novel anti-inflammatory agent for sarcoidosis. Sarcoidosis Vasc Diffuse Lung Dis. 2023;40:e2023011. 10.36141/svdld.v40i1.14396. 36975051 10.36141/svdld.v40i1.14396 PMC10099656

[23] Grunewald J, Grutters JC, Arkema EV, et al. Sarcoidosis Nat Rev Dis Primers. 2019;5:45. 10.1038/s41572-019-0096-x. 31273209 10.1038/s41572-019-0096-x

[24] Drent M, Crouser ED, Grunewald J. Challenges of Sarcoidosis and its management. N Engl J Med. 2021;385:1018–32. 10.1056/NEJMra2101555. 34496176 10.1056/NEJMra2101555

[25] Janssen MTHF, Landewé RBM, Post MC, et al. Organ involvement and assessment in sarcoidosis. Curr Opin Pulm Med. 2023;29:485–92. 10.1097/MCP.0000000000000997. 37461850 10.1097/MCP.0000000000000997

[26] Judson MA, Baughman RP, Costabel U, et al. Efficacy of infliximab in extrapulmonary sarcoidosis: results from a randomised trial. Eur Respir J. 2008;31:1189. 10.1183/09031936.00051907. 18256069 10.1183/09031936.00051907

[27] Evans M, Sharma O, LaBree L, Smith RE, Rao NA. Differences in clinical findings between caucasians and African americans with biopsy-proven sarcoidosis. Ophthalmology. 2007;114:325–33. 10.1016/j.ophtha.2006.05.074. 17123620 10.1016/j.ophtha.2006.05.074

[28] Crouser ED, Maier LA, Wilson KC, et al. Diagnosis and detection of Sarcoidosis. An official American thoracic Society Clinical Practice Guideline. Am J Respir Crit Care Med. 2020;201:e26. 10.1164/rccm.202002-0251ST. 32293205 10.1164/rccm.202002-0251ST PMC7159433

[29] Sève P, Jamilloux Y, Tilikete C, et al. Ocular Sarcoidosis. Semin Respir Crit Care Med. 2020;41:673–88. 10.1055/s-0040-1710536. 32777852 10.1055/s-0040-1710536

[30] Mochizuki M, Smith JR, Takase H, et al. Revised criteria of International Workshop on Ocular Sarcoidosis (IWOS) for the diagnosis of ocular sarcoidosis. Br J Ophthalmol. 2019;103:1418. 10.1136/bjophthalmol-2018-313356. 10.1136/bjophthalmol-2018-313356 30798264

[31] Matsou A, Tsaousis TT. Management of chronic ocular sarcoidosis: challenges and solutions. Clin Ophthalmol. 2018;12:519–32. 10.2147/OPTH.S128949. 29593377 10.2147/OPTH.S128949 PMC5863717

[32] Haimovic A, Sanchez M, Judson MA, Prystowsky S. Sarcoidosis: a comprehensive review and update for the dermatologist: part I. cutaneous disease. J Am Acad Dermatol. 2012;66:699e1. 10.1016/j.jaad.2011.11.965. 10.1016/j.jaad.2011.11.965 22507585

[33] Marchell RM, Judson MA. Cutaneous sarcoidosis. Semin Respir Crit Care Med. 2010;31:442–51. 10.1055/s-0030-1262212. 10.1055/s-0030-1262212 20665394

[34] Yatsynovich Y, Dittoe N, Petrov M, et al. Cardiac sarcoidosis: a review of Contemporary challenges in diagnosis and treatment. Am J Med Sci. 2018;355:113–25. 10.1016/j.amjms.2017.08.009. 29406038 10.1016/j.amjms.2017.08.009

[35] Murtagh G, Laffin LJ, Patel KV, et al. Improved detection of myocardial damage in sarcoidosis using longitudinal strain in patients with preserved left ventricular ejection fraction. Echocardiography. 2016;33:1344–52. 10.1111/echo.13281. 27677642 10.1111/echo.13281 PMC5523448

[36] Kouranos V, Sharma R. Cardiac sarcoidosis: state-of-the-art review. Heart. 2021;107:1591–9. 10.1136/heartjnl-2019-316442. 33674355 10.1136/heartjnl-2019-316442

[37] Masri SC, Bellumkonda L. Sarcoid Heart Disease: an update on diagnosis and management. Curr Cardiol Rep. 2020;22:177. 10.1007/s11886-020-01429-4. 33119794 10.1007/s11886-020-01429-4

[38] Terushkin V, Stern BJ, Judson MA, et al. Neurosarcoidosis: presentations and management. Neurologist. 2010;16:2. 10.1097/NRL.0b013e3181c92a72. 20065791 10.1097/NRL.0b013e3181c92a72

[39] Menninger MD, Amdur RJ, Marcus RB Jr. Role of radiotherapy in the treatment of neurosarcoidosis. Am J Clin Onco. 2003;26:e115. 10.1097/01.COC.0000077933.69101.5D. 10.1097/01.COC.0000077933.69101.5D 12902908

[40] Werner J, Rivera N, Grunewald J, et al. HLA-DRB1 alleles associate with hypercalcemia in sarcoidosis. Respir Med. 2021;187:106537. 10.1016/j.rmed.2021.106537. 34325227 10.1016/j.rmed.2021.106537

[41] Rastelli F, Baragetti I, Buzzi L, et al. Renal involvement in sarcoidosis: histological patterns and prognosis, an Italian survey. Sarcoidosis Vasc Diffuse Lung Dis. 2021;38:e2021017. 10.36141/svdld.v38i3.11488. 34744417 10.36141/svdld.v38i3.11488 PMC8552569

[42] Sharma A, Kadakia J, Sharma O. Gastrointestinal Sarcoidosis. Sem Respir Med. 1992;6:442. 10.1055/s-2007-1006293.

[43] Cremers JP, Drent M, Baughman RP et al. Therapeutic approach of hepatic sarcoidosis. Curr Opin Pulm Med;18:472–82. 10.1097/MCP.0b013e3283541626. 10.1097/MCP.0b013e3283541626 22617809

[44] Hangül M, Köse M, Pekcan S, Ersoy A, Unal G, Caglar HT. Childhood sarcoidosis in the middle Anatolia of Turkey. Pediatr Pulmonol. 2023. 10.1002/ppul.26564. 37341617 10.1002/ppul.26564

[45] Kaufman K, Becker ML. Distinguishing Blau Syndrome from systemic sarcoidosis. Curr Allergy Asthma Rep. 2021;21:10. 10.1007/s11882-021-00991-3. 33560445 10.1007/s11882-021-00991-3 PMC9762981

